# Designing efficient hybrid strategies for information spreading in scale-free networks

**DOI:** 10.1098/rsos.180117

**Published:** 2018-08-01

**Authors:** Shuangyan Wang, Wuyi Cheng, Yang Hao

**Affiliations:** School of Engineering and Technology, China University of Geosciences, Beijing, People's Republic of China

**Keywords:** complex network, scale-free network, spreading strategy, hybrid strategy, Monte Carloexperiments

## Abstract

Designing a spreading strategy is one of the critical issues strongly affecting spreading efficiency in complex networks. In this paper, to improve the efficiency of information spreading in scale-free networks, we propose four hybrid strategies by combining two basic strategies, i.e. (i) the LS (in which information is preferentially spread from the large-degree vertices to the small-degree ones), and (ii) the SL (in which information is preferentially spread from the small-degree vertices to the large-degree ones). The objective in combining the two basic LS and SL strategies is to fully exploit the advantages of both strategies. To evaluate the spreading efficiency of the proposed four hybrid strategies, we first propose an information spreading model. Then, we introduce the details of the proposed hybrid strategies that are formulated by combining LS and SL. Third, we build a set of scale-free network structures by differently configuring the relevant parameters. In addition, finally, we conduct various Monte Carlo experiments to examine the spreading efficiency of the proposed hybrid strategies in different scale-free network structures. Experimental results indicate that the proposed hybrid strategies are effective and efficient for spreading information in scale-free networks.

## 1. Introduction

In the context of network theory, a complex network is a graph (network) with non-trivial topological features—features that do not occur in simple networks such as lattices or random graphs but often occur in graphs modelling real systems [1–3]. Most social, biological and technological networks display substantial non-trivial topological features, with patterns of connections between their elements that are neither purely regular nor purely random [4]. In real life, the social relationships among people can also be seen as a complex network with non-trivial topological features [5–7]. In addition, the social relationships among people are always the channels by which information is delivered to people. The communications and interactions among people can be seen as a complex network. One of the typical complex networks is a scale-free network [8]. In addition, most research work indicates that social networks typically exhibit scale-free network properties [9–11]. Information spreading in scale-free networks has been intensively studied [12–22]. Most research work primarily focuses on the paths, methods, strategies and processes of information spreading. For instance, Salvatore Cuomo *et al.* [23,24] proposed the Integrate & Fire (IF) network to analyse the information sharing and typical behaviours between visitors. In their approach, the visitor is modelled as a computational neuron, which is connected with his friends by means of synapses (i.e. connections), representing friendship and/or common interests among them. The key objective in developing optimized paths and effective spreading strategies is to improve the efficiency of spreading [25–29]. For example, Dai *et al.* [5] collected Internet data and analysed the process of broadcasting information in social networks. Song *et al.* [6] studied the factors influencing information spread in online social networks. Wu *et al.* [7] studied the influence of trust in the spreading of information and validated their analysis in homogeneous (random network) and heterogeneous (scale-free network) networks. There are two typical information spreading strategies reported in previous work. The first one, denoted LS for short, emphasized that the information could be significantly diffused when all vertices in a network spread the information by preferentially selecting the large-degree (influential vertices) vertices [26,30]. The second one, denoted SL for short, emphasized that the information could be significantly diffused when all vertices in a network spread the information by preferentially selecting the small-degree vertices [27,29]. It has been widely learned that the large-degree vertices in a scale-free network are of greater diffusions because they have more connections, and it typically costs much more spreading time for small-degree vertices owing to their fewer connections. Thus, the LS strategy effectively exploits the diffusion of large-degree vertices, while the SL strategy reduces the consumption of spreading small-degree vertices. In this paper, to improve the efficiency of spreading information, we propose four hybrid strategies by combining the two strategies LS and SL via fully exploiting the advantages of both strategies. To evaluate the spreading efficiency of the proposed four hybrid strategies, we first propose an information spreading model. Then, we introduce the details of the proposed hybrid strategies that are formulated by combining LS and SL. Third, we build a set of scale-free network structures by differently configuring the relevant parameters. Fourth, we simulate the information spreading when using the four hybrid strategies and the two strategies (i.e. the LS and SL) in scale-free networks with different structures; and for each simulation, we conduct the Monte Carlo experiment to calculate the convergence time of the information spreading. Finally, we examine the spreading efficiency of the proposed hybrid strategies in different scale-free network structures. The contributions of this work can be summarized as follows: (i) we propose an information spreading model based on multi-agent modelling; and (ii) we propose four hybrid strategies, which are formulated by combining the LS and SL to enhance the spreading efficiency. The rest of this paper is organized as follows: §2 gives a brief introduction to the problem that is to be addressed in this paper; §3 describes the proposed information spreading model and the hybrid strategies in detail; in addition, §3 also gives an introduction to the experimental process for evaluating the spreading efficiency; and §§4 and 5 present and discuss the results of the experiments, respectively. Finally, §6 draws several conclusions.

## 2. Problem statement

In this section, we will give a brief introduction to the problem focused on in this paper.

### 2.1. The boundaries and combinations of hybrid strategies

**Figure 1.**
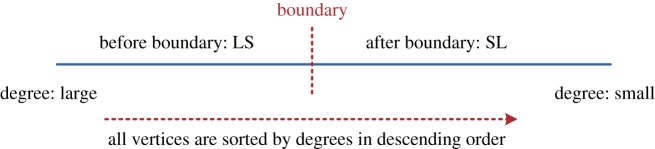
A simple illustration of a hybrid strategy which is formulated by combining the LS and SL.

The key objective in developing hybrid strategies by combining the LS and SL is to exploit the advantages of both the LS and SL. In combining the LS and SL, we first need to determine the boundaries of hybrid strategies that separate the use of LS and SL (figure 1). Moreover, we also need to clarify the best combinations of LS and SL in terms of spreading efficiency in different network structures. For instance, vertices that are located before the boundary, spread information by using the LS (or SL), and vertices that are located after the boundary, spread the information by using the SL (or LS).

### 2.2. The network structures (scenes) of information spreading

The network structures (also called the spreading scenes) have significant influences on the spreading efficiency [31]. That is, the spreading efficiency of the spreading with different strategies depends on realistic scenes. Thus, we first need to control and change the relevant parameters of network structures and then evaluate our hybrid strategies in different network structures.

## 3. The proposed method

### 3.1. The proposed information spreading model

In this subsection, we give an introduction to the assumptions and mechanisms in our proposed information spreading model.

(i) *Assumption 1*: some vertices can be informed randomly at the beginning of simulations.
  Vertices that are informed randomly at the beginning of the simulation are called the initial informed vertices (IIVs for short). Those IIVs diffuse the information at the beginning of simulations. In addition, the information will then be diffused according to the spreading mechanism and the spreading strategies.
(ii) *Assumption 2*: uninformed vertices become informed vertices after receiving information.
  In our spreading model, to simplify the spreading process, we assume that the uninformed vertices will become the informed vertices immediately after receiving the information.
(iii) *Assumption 3*: there is a delay period before the informed vertices spread the information.
  We consider that the people who have known the information cannot spread the information immediately and that the waiting time before they spread the information is varied for different people. Therefore, there is a delay period before the informed vertices spread the information, and the delay time conforms to the uniform distribution in our spreading model.
(iv) *Assumption 4*: informed vertices spread the information based on different probabilities.
  We consider that the spreading probabilities of people who have known the information are different. Thus, we assume the probability conforms to the uniform distribution of 0–1.
(v) *Assumption 5*: all informed vertices spread the information.
  To preferably evaluate the spreading efficiency of different strategies, we assume that all informed vertices will spread the information. In addition, according to assumptions 3 and 4, we can learn that all informed vertices will spread information with different delay times and spreading probabilities.

**Figure 2.**
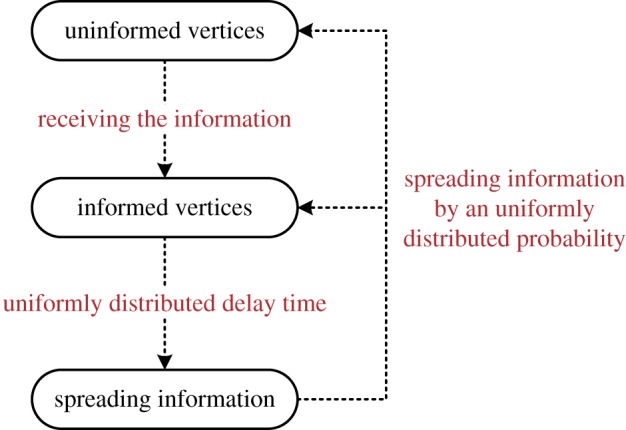
A simple flowchart of the proposed information spreading model.

Based upon the above assumptions, an information spreading model is proposed in this paper; see a simple flowchart of the proposed model in figure 2. At the beginning of the experiment, all vertices are initially set as uninformed vertices; meanwhile, several uninformed vertices are informed randomly (IIVs), which are the most original informed vertices. Then, the vertices that have known the information become the informed vertices, which have the opportunity and qualifications to diffuse the information. After a random delay period, the informed vertices can spread information to connected vertices conforming to a random probability.

### 3.2. The proposed hybrid strategies

In this subsection, we will give an introduction to the determinations of boundaries and the combinations for the proposed hybrid strategies.

#### 3.2.1. The boundaries of hybrid strategies.

The first critical step in developing hybrid strategies is to determine the boundaries that separate the LS and SL. In this paper, we first sort all vertices according to their degrees in descending order, and then, we select the following two boundaries: (i) we select 20% of the population (the network size) according to the Pareto principle. The Pareto Principle (80/20 rule) commonly occurs in many social phenomena and scale-free networks. According to the Pareto principle, there are 20% of vertices that have approximately 80% of the resources (connections) in the network. We think the 20% of the population could be a kind of boundary; and (ii) as the mean of the population, we think 50% of the population (the network size) could be a kind of boundary.

#### 3.2.2. The combinations of hybrid strategies.

**Figure 3.**
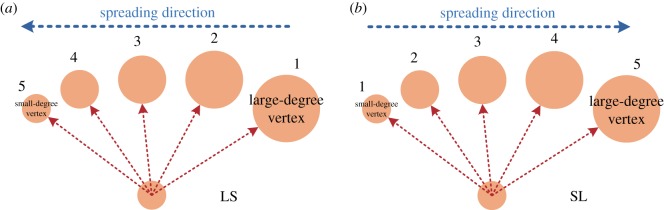
The spreading directions of (*a*) LS and (*b*) SL . (*a*) Vertices spread the information by preferentially selecting the large-degree nodes. (*b*) Vertices spread the information by preferentially selecting the small-degree nodes. The diameter of a vertex represents the degree of the vertex. The numbers located on the top of the vertices represent the spreading serial numbers.

According to the LS and SL, there are two kinds of spreading directions for vertices: (i) information is diffused from large-degree connected vertices to small-degree connected vertices (LS for short; figure 3*a*); and (ii) information is diffused from small-degree connected vertices to large-degree connected vertices (SL for short; figure 3*b*).

**Figure 4.**
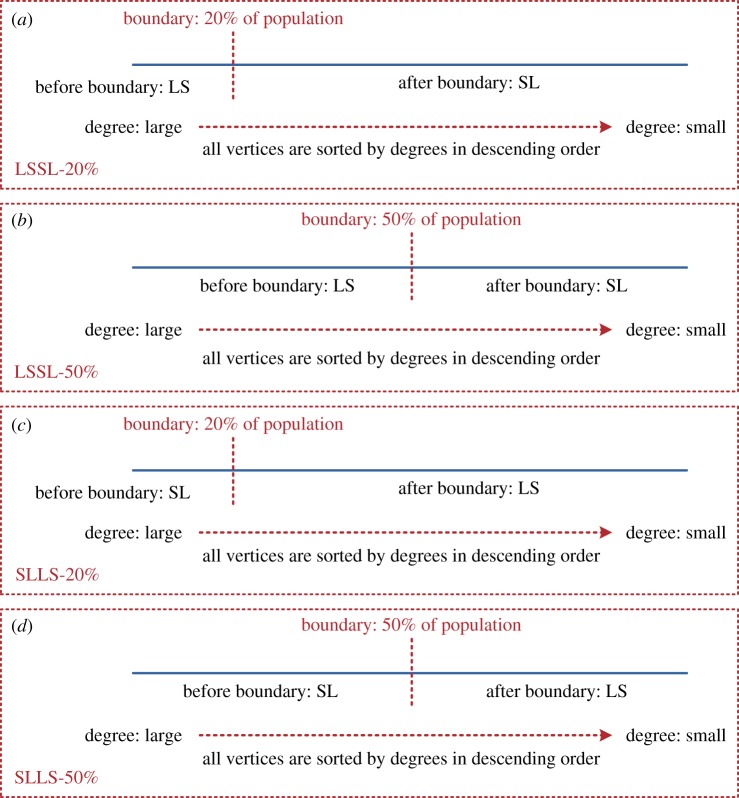
The four proposed hybrid strategies (i.e. four combinations of LS and SL).

By combining different spreading directions and the boundaries, we can obtain four hybrid strategies (figure 4 and table 1).

**Table 1.** Boundaries and combinations of different hybrid strategies.

			spreading direction	
hybrid strategy	abbreviation	boundary	before boundary	after boundary
HS1	LSSL-20%	20% of population	LS	SL
HS2	LSSL-50%	50% of population	LS	SL
HS3	SLLS-20%	20% of population	SL	LS
HS4	SLLS-50%	50% of population	SL	LS

‘LSSL-20%’: the 20% of the population is the boundary in this hybrid strategy, the vertices before the boundary spread information by using LS and the vertices after the boundary spread information by using SL.

‘LSSL-50%’: the 50% of the population is the boundary in this hybrid strategy, the vertices before the boundary spread information by using LS and the vertices after the boundary spread information by using SL.

‘SLLS-20%’: the 20% of the population is the boundary in this hybrid strategy, the vertices before the boundary spread information by using SL and the vertices after the boundary spread information by using SL.

‘SLLS-50%’: the 50% of the population is the boundary in this hybrid strategy, the vertices before the boundary spread information by using SL and the vertices after the boundary spread information by using LS.

### 3.3. Design of numerical experiments with different strategies in different network structures

We perform some numerical experiments with different settings of parameters to evaluate our hybrid strategies. In this subsection, we give an introduction to the settings of experiments and the experimental process.

#### 3.3.1. The building of different scale-free network structures for testing.

In this paper, we will evaluate the spreading efficiency of our hybrid strategies in different scale-free network structures. We use the software Anylogic [32], which is a multi-agent modelling platform, to build different scale-free networks. In addition, there are two parameters needed to build the scale-free network structure, i.e. (i) the size of the network and (ii) the number of hubs (the vertices have many more connections than others) in the network. We could build various scale-free network structures by changing the settings of the above two parameters.

#### 3.3.2. The experiment process.

According to the assumptions that we have introduced in §3.1, we assume that there are 100 vertices that can be informed randomly at the beginning of simulations. The settings of the sizes of the population and the number of hubs are listed in table 2.

**Table 2.** Configurations of the relevant two parameters in the experiments.

number	size of population	number of hubs
NS1	500	two
NS2		three
NS3		four
NS4	1000	two
NS5		three
NS6		four
NS7	2000	four
NS8		six
NS9		eight

**Figure 5.**
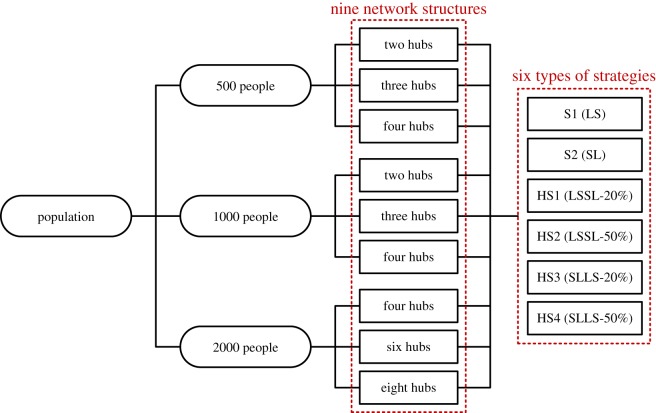
The experimental configurations.

We employ three sizes of population, i.e. 500 people, 1000 people and 2000 people. In addition, correspondingly, we set the numbers of hubs for each population: we set the same numbers of hubs for the experiments with 500 people and 1000 people, i.e. two hubs, three hubs and four hubs, and we set the number of hubs for the same percentages (0.2%, 0.3% and 0.4%) in the experiment with 2000 people, i.e. four hubs, six hubs and eight hubs. We have performed the pre-experiments, which indicate that the settings of the sizes of the population and the number of hubs are reliable. With the use of the above settings, we obtain nine different network structures. There are six different strategies (i.e. the LS, SL and four hybrid strategies; figure 5).

To investigate the spreading efficiency of our hybrid strategies in different scenes, we use the Monte Carlo method in Anylogic to perform our experiments. Monte Carlo experiments are a broad class of computational algorithm that rely on repeated random sampling to obtain numerical results. Their essential idea is using randomness to solve problems that might be deterministic in principle [33]. In our information spreading model, we consider three typical affecting factors of the information spreading: (i) the initial randomly informed vertices, (ii) the delay period before spreading for each vertex, and (iii) the information spreading probability for each vertex. The above three factors can be affected by the random seeds of simulations, and different random seeds may create different information spreading processes. We use the Monte Carlo experiments to reduce the influence of random seeds on experimental results. We use a defined random generator to generate the random numbers from 0 to 1000 (excluding 1000) and repeat the simulation 1000 times for each experiment.

## 4. Results

There is a convergence time of each simulation in Monte Carlo experiments; and when all simulations are finished, the Monte Carlo experiment in Anylogic [32] can automatically generate a frequency distribution of convergence time. We set 0.1 as the initial interval of the frequency distribution, and the Monte Carlo experiments can automatically detect the interval scope of the frequency distribution according to the convergence time of each simulation. In addition, we set 300 as the number of intervals in the frequency distribution. We use a defined random generator to generate the random numbers ranging from 0 to 1000 (excluding 1000) and repeat the simulation 1000 times in each Monte Carlo experiment.

**Figure 6.**
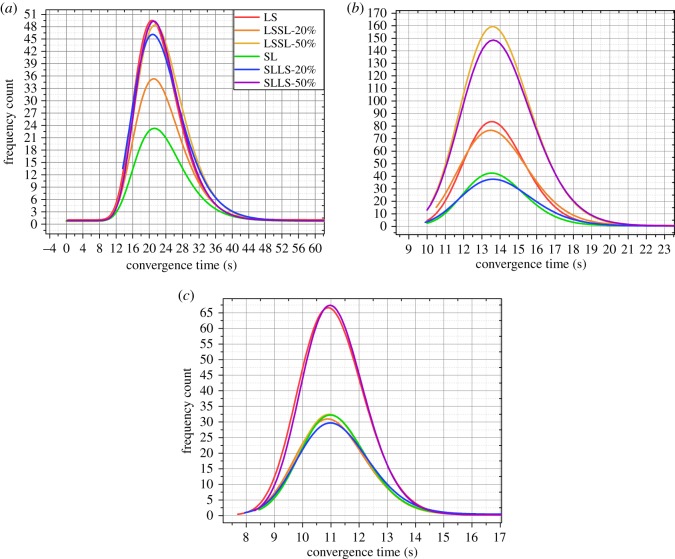
The results of the experiments with 500 people. (*a*) The results of the experiment with 500 people and two hubs. (*b*) The results of the experiment with 500 people and three hubs. (*c*) The results of the experiment with 500 people and four hubs. LS expresses a basic strategy in which all vertices spread the information by preferentially selecting the large-degree connected vertices. SL expresses a basic strategy in which all vertices spread the information by preferentially selecting the small-degree connected vertices. LSSL-20% expresses a hybrid strategy whose boundary is 20% of the population, and the vertices before the boundary spread the information according to LS; the vertices after the boundary spread the information according to SL. LSSL-50% expresses a hybrid strategy whose boundary is 50% of the population, and the vertices before the boundary spread the information according to LS; the vertices after the boundary spread the information according to SL. SLLS-20% expresses a hybrid strategy whose boundary is 20% of the population, and the vertices before the boundary spread the information according to SL; the vertices after the boundary spread the information according to LS. SLLS-50% expresses a hybrid strategy whose boundary is 50% of the population, and the vertices before the boundary spread the information according to SL; the vertices after the boundary spread the information according to LS.

**Figure 7.**
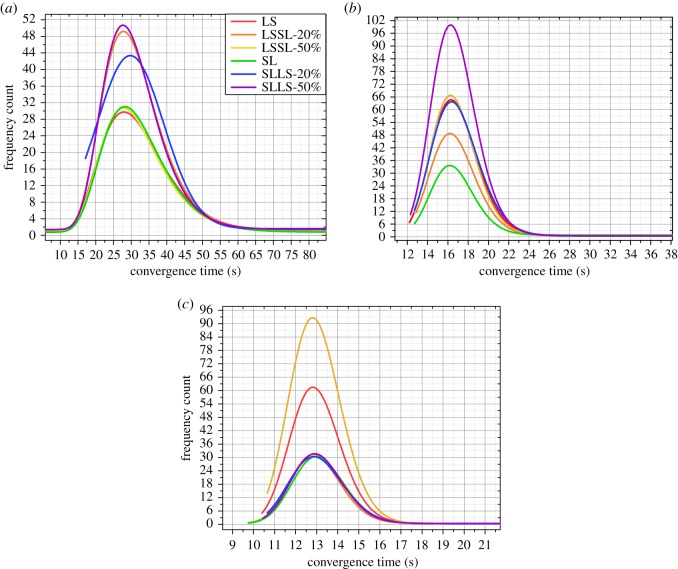
The results of the experiments with 1000 people. (*a*) The results of the experiment with 1000 people and two hubs. (*b*) The results of the experiment with 1000 people and three hubs. (*c*) The results of the experiment with 1000 people and four hubs. LS expresses a basic strategy in which all vertices spread the information by preferentially selecting the large-degree connected vertices. SL expresses a basic strategy in which all vertices spread the information by preferentially selecting the small-degree connected vertices. LSSL-20% expresses a hybrid strategy whose boundary is 20% of the population, and the vertices before the boundary spread the information according to LS; the vertices after the boundary spread the information according to SL. LSSL-50% expresses a hybrid strategy whose boundary is 50% of the population, and the vertices before the boundary spread the information according to LS; the vertices after the boundary spread the information according to SL. SLLS-20% expresses a hybrid strategy whose boundary is 20% of the population, and the vertices before the boundary spread the information according to SL; the vertices after the boundary spread the information according to LS. SLLS-50% expresses a hybrid strategy whose boundary is 50% of the population, and the vertices before the boundary spread the information according to SL; the vertices after the boundary spread the information according to LS.

**Figure 8.**
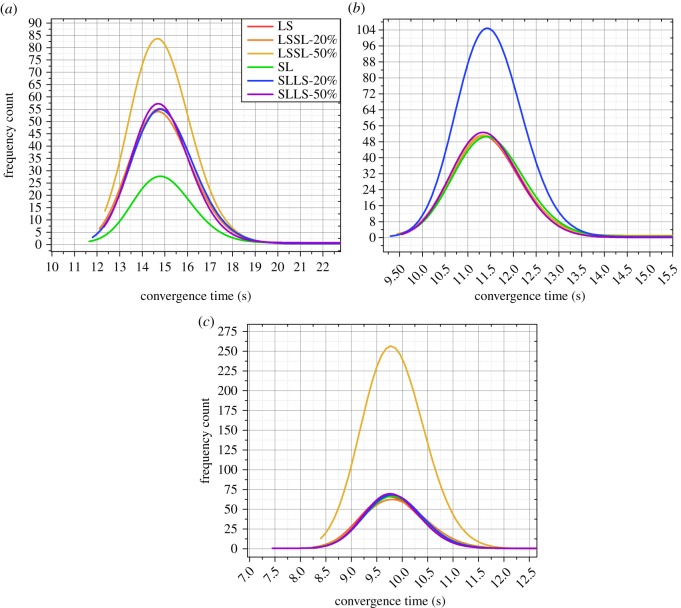
The results of the experiments with 2000 people. (*a*) The results of the experiment with 2000 people and four hubs. (*b*) The results of the experiment with 2000 people and six hubs. (*c*) The results of the experiment with 2000 people and eight hubs. LS expresses a basic strategy in which all vertices spread the information by preferentially selecting the large-degree connected vertices. SL expresses a basic strategy in which all vertices spread the information by preferentially selecting the small-degree connected vertices. LSSL-20% expresses a hybrid strategy whose boundary is 20% of the population, and the vertices before the boundary spread the information according to LS; the vertices after the boundary spread the information according to SL. LSSL-50% expresses a hybrid strategy whose boundary is 50% of the population, and the vertices before the boundary spread the information according to LS; the vertices after the boundary spread the information according to SL. SLLS-20% expresses a hybrid strategy whose boundary is 20% of the population, and the vertices before the boundary spread the information according to SL; the vertices after the boundary spread the information according to LS. SLLS-50% expresses a hybrid strategy whose boundary is 50% of the population, and the vertices before the boundary spread the information according to SL; the vertices after the boundary spread the information according to LS.

**Figure 9.**
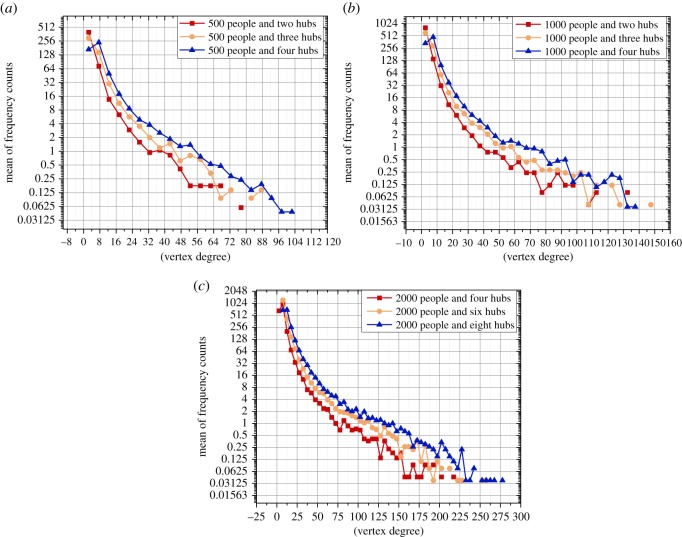
The degree distributions of different network structures. (*a*) The degree distributions of the networks with 500 people and two hubs, three hubs and four hubs, respectively; (*b*) the degree distributions of the networks with 1000 people and two hubs, three hubs and four hubs, respectively; and (*c*) the degree distributions of the networks with 2000 people and four hubs, six hubs and eight hubs, respectively.

Then we perform some pre-treatments: we fit the frequency distribution of each strategy in an experiment with a network structure; and put the fitting curves of all spreading strategies in a figure. The average value of each interval of the frequency distribution is used as the *x*-axis in figures 6–9.

### 4.1. Experimental results for 500 people

We integrate all fitting curves into a figure for the convenience of comparisons. Figure 6*a* illustrates the results of the experiment with 500 people and two hubs, figure 6*b* illustrates the results of the experiment with 500 people and three hubs, and figure 6*c* illustrates the results of the experiment with 500 people and four hubs. As shown in figure 6, in the experiment with 500 people and two hubs, there are slight differences between the spreading efficiency when using ‘LSSL-50%’, ‘SLLS-50%’ and the ‘LS’; however, the spreading using the three kinds of strategies is more efficient than when using the other strategies. In the experiment with 500 people and three hubs, the spreading using the ‘LSSL-50%’ is the most efficient, and there are slight differences between the spreading efficiency when using ‘LSSL-50%’ and ‘SLLS-50%’. In addition, in the experiment with 500 people and four hubs, there are slight differences between the ‘SLLS-50%’ and ‘LS’; however, the spreading using the two kinds of strategies is more efficient than that when using the other strategies.

### 4.2. Experimental results for 1000 people

Figure 7*a* illustrates the results of the experiment with 1000 people and two hubs, figure 7*b* illustrates the results of the experiment with 1000 people and three hubs and figure 7*c* illustrates the results of the experiment with 1000 people and four hubs. As shown in figure 7, we can find that in the experiment with 1000 people and two hubs, the spreading when using ‘LSSL-20%’, ‘SLLS-20%’ and ‘SLLS-50%’ is efficient; the spreading when using ‘SLLS-50%’ is the most efficient; and there are slight differences between the spreading efficiency when using ‘SLLS-50%’ and ‘LSSL-20%’. The spreading when using ‘SLLS-50%’ is the most efficient in the experiment with 1000 people and three hubs. The spreading when using ‘LSSL-50%’ is the most efficient in the experiment with 1000 people and four hubs.

### 4.3. Experimental results for 2000 people

Figure 8*a* illustrates the results of the experiment with 2000 people and four hubs, figure 8*b* illustrates the results of the experiment with 2000 people and six hubs, and figure 8*c* illustrates the results of the experiment with 2000 people and eight hubs. As shown in figure 8, in the experiment with 2000 people and four hubs, the spreading when using ‘LSSL-50%’ is the most efficient, and there are slight differences between the spreading efficiency when using ‘SLLS-50%’, ‘LSSL-20%’, ‘SLLS-20%’ and ‘LS’. In the experiment with 2000 people and six hubs, the spreading when using ‘SLLS-20%’ is the most efficient, and there are slight differences between the spreading efficiency when using the other strategies. In the experiment with 2000 people and eight hubs, the spreading when using ‘LSSL-50%’ is the most efficient, and there are slight differences between the spreading efficiency when using other strategies.

## 5. Discussion

### 5.1. Spreading efficiency of hybrid strategies

The spreading efficiency of our hybrid strategies has been illustrated in figures 6–8. Those results indicated that the spreading when using our hybrid strategies is effective and efficient. The most efficient hybrid strategy is different for different network structures because of the influences of network structures.

### 5.2. Effects of different network structures on spreading efficiency

We have created different network structures by setting different (i) sizes of population and (ii) numbers of hubs. As shown in figure 9, the distributions of vertices' degrees when setting different parameters (i.e. the size of the population and the number of hubs) are varied. In our opinion, we think that the differences in network structures could strongly affect the spreading efficiency. We find that a significant improvement in the spreading efficiency when using the best efficient hybrid strategy can be achieved in some experiments, while in other experiments, the improvement in the spreading efficiency when using the best efficient hybrid strategy is not significant. For instance, in the experiment with 500 people and two hubs, there are slight differences between the spreading efficiency of using ‘LSSL-50%’, ‘SLLS-50%’ and the ‘LS’; however, in the experiment with 2000 people and eight hubs, the spreading using ‘LSSL-50%’ is obviously the most efficient. We think that the network structure strongly affects the improvement of the spreading efficiency when using the best efficient hybrid strategy. We also find that the network structures could affect both the boundary and combination of the most efficient hybrid strategy. For example, in the experiment with 2000 people and six hubs, the spreading using ‘SLLS-20%’ is the most efficient; in the experiment with 2000 and four hubs, the spreading using ‘LSSL-50%’ is the most efficient; and in the experiment with 1000 and three hubs, the spreading using ‘SLLS-50%’ is the most efficient.

### 5.3. Determination of the most efficient hybrid strategies in different network structures

According to the experimental results illustrated in figures 6–8, we have found that the spreading using the hybrid strategies in which the boundary is 50% of the population is the most efficient in almost all experiments, except for the experiment with 2000 people and six hubs. In the experiment with 2000 people and six hubs, the spreading using ‘SLLS-20%’ is the most efficient. Moreover, the combined spreading directions of the best hybrid strategy are different in different experiments. For example, the spreading using ‘SLLS-50%’ is the most efficient in the experiment with 1000 people and three hubs; however, the spreading using ‘LSSL-50%’ is the most efficient in the experiment with 1000 people and four hubs. For the results where the combined spreading directions of the best hybrid strategy are different in different experiments, we think that the combined spreading directions of the best hybrid strategy are perhaps affected by the network structures or some other influential factors. In this paper, all vertices are sorted by degrees in descending order, which is the first step in developing our hybrid strategies. Thus, the first 50% of vertices are the larger-degree vertices, which are capable of diffusing much more strongly because of much more connections with them. When the first 50% of vertices spread the information by using the LS, the first 50% of the larger-degree vertices diffuse the information to the connected larger-degree vertices to exploit the diffusion capability of connected larger-degree vertices. Thus, we think that it may need more large-degree vertices to exploit the diffusion capability in the network where the spreading using ‘LSSL-50%’ is the most efficient. When the first 50% of vertices spread the information by using the SL, the first 50% larger-degree vertices diffuse the information to the connected smaller-degree vertices to reduce the time consumed for spreading to small-degree vertices in the network because of many more connections with the first 50% of the larger-degree vertices. Thus, we think that it may need the first 50% larger-degree vertices to exploit the diffusion for spreading the information to the small-degree vertices in the network where the spreading using ‘SLLS-50%’ is the most efficient. For the result that 50% of the population is the boundary in almost all the experiments, we think that the boundary of the best hybrid strategy could also be affected by the network structures or some other influential factors. We think that in the efficient spreading using ‘SLLS-20%’, 20% of the population (the first 20% large-degree vertices) is adequate for reducing the spreading time consumed to spread small-degree vertices. For the efficient spreading using ‘LSSL-50%’, we think that the first 50% of the population is needed to diffuse the information to the connected larger-degree vertices to exploit the diffusion capability of large-degree vertices in the network.

## 6. Conclusion

In this paper, we have proposed four efficient hybrid strategies for spreading information in scale-free networks. The essential idea behind developing our hybrid strategies is to exploit the advantages of two basic strategies, i.e. the LS and SL. The crucial first step in developing our hybrid strategies is the determination of the boundaries that separate the LS and SL. In our work, the boundaries separating the LS and SL are configured according to the Pareto principle and the mean of the population. By configuring different boundaries and combinations of the LS and SL, we have obtained four hybrid strategies. To evaluate the spreading efficiency of the proposed four hybrid strategies in different network structures, we have first built an information spreading model and a set of different network structures by setting different parameters (i.e. the size of the population and the number of hubs). We have then conducted various Monte Carlo experiments with different scale-free network structures. The experimental results indicated that the proposed hybrid strategies are effective and efficient for information spreading in scale-free networks. Moreover, we have also found that the network structure can significantly affect the improvement in the spreading efficiency, the combinations and the boundary of the most efficient hybrid strategy. Thus, we may first need to perform some pre-experiments to determine the most suitable and efficient hybrid strategy for a specific, realistic network structure and employ the determined hybrid strategy in a practical application.

## Data accessibility

Our data are deposited at Dryad: (https://doi.org/10.5061/dryad.kj2d3) [34].
